# Lifestyle and Cardiovascular Risk Factors by Educational Attainment in a Mediterranean Setting: A Cross-Sectional Analysis of a Population-Based Sample

**DOI:** 10.5334/gh.1574

**Published:** 2026-07-14

**Authors:** Dolores Álamo-Junquera, Carles Vilaplana-Carnerero, Constança Pagès-Fernández, Diana Toledo, Núria Soldevila, Alba Tor-Roca, Àngela Domínguez, María Grau

**Affiliations:** 1 Catalan Institute of Health, Sabadell, Spain; 2 Department of Medicine, School of Medicine and Health Sciences, University of Barcelona, Barcelona, Spain; 3 Service for the Promotion of Quality and Bioethics, General Directorate of Health Planning and Regulation, Department of Health, Government of Catalonia, Barcelona, Spain; 4 Biomedical Research Consortium in Epidemiology and Public Health (CIBERESP), Madrid, Spain; 5 Jordi Gol i Gurina Primary Health Care Research Institute Foundation (IDIAPJGol), Barcelona, Spain; 6 Research Institute for Nutrition and Food Safety (INSA-UB), Santa Coloma de Gramenet, Spain; 7 Instituto de Salud Global de Barcelona (ISGlobal), Barcelona, Spain

**Keywords:** Educational attainment, Cardiovascular risk factors, Socioeconomic inequalities, Lifestyle behaviors, Sex differences

## Abstract

**Background:**

Socioeconomic inequalities in cardiovascular risk persist despite declining mortality. We examined the prevalence of cardiovascular risk factors and lifestyle behaviors across educational levels in men and women from a Mediterranean population.

**Methods:**

Cross-sectional analysis of adults aged 35–74 years from a population-based sample in north-eastern Spain. Risk factors and lifestyle were assessed with standardized measurements and validated questionnaires. Educational attainment was categorized as primary, secondary, or university. Analyses were sex-stratified; age-adjusted linear and logistic regression models were applied.

**Results:**

We included 943 adults (52.3% women). Among men, secondary and university education (vs. primary) were associated with lower LDL-cholesterol [β –11.76 (95% CI –21.68 to –1.83) and –17.83 (–28.03 to –7.63) mg/dL], lower odds of hypercholesterolemia [OR = 0.43 (0.20–0.90) for both], smoking [OR = 0.57 (0.33–0.97) and 0.53 (0.30–0.95)], and higher odds of high Mediterranean diet adherence [OR = 2.08 (1.04–4.16) and 3.05 (1.51–6.14)]. Among women, secondary and university education (vs. primary) were associated with lower body mass index [β –1.81 (–2.85 to –0.77) and –2.52 (–3.63 to –1.40) kg/m^2^] and lower odds of overweight/obesity [OR = 0.47 (0.29–0.76) and 0.34 (0.20–0.56)]. Lower odds of hypertension [OR = 0.44 (0.22–0.87)] and high Mediterranean diet adherence [OR = 2.14 (1.12–4.09)] were also more likely in university-educated women.

**Conclusions:**

Higher educational attainment was associated with healthier lifestyles and more favorable cardiometabolic profiles. Educational gradients were broader in women—spanning adiposity, blood pressure, glycemia, and lipidemia—whereas, in men, they mainly affected lipidemia and diet.

## Graphical Abstract

**Figure:**
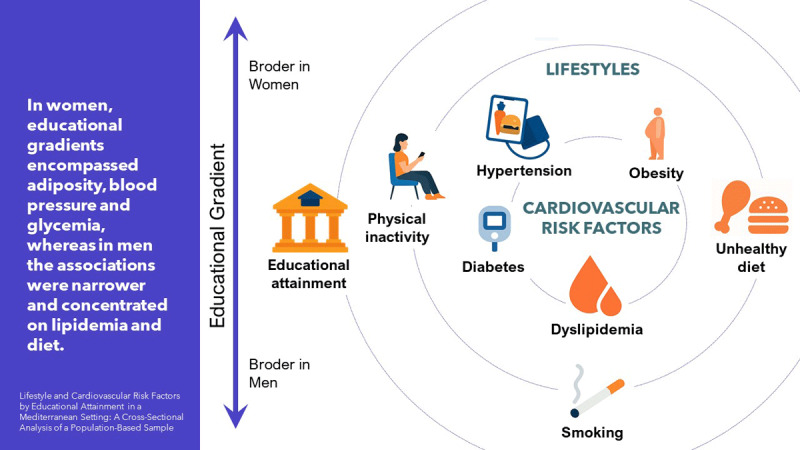


## Introduction

Cardiovascular diseases remain the leading cause of morbidity and mortality worldwide, despite substantial declines in mortality rates in high-income countries over recent decades (1). Approximately half of this reduction has been attributed to evidence-based clinical treatments, while the other half is explained by improvements in lifestyle factors such as diet, physical activity, and smoking cessation (2). However, these improvements have not been equally distributed, and socioeconomic inequalities in cardiovascular risk persist (3).

Evidence from this Mediterranean population indicates a high prevalence of cardiovascular risk factors, with hypertension affecting 19% of women and 36% of men, hypercholesterolemia 74% and 80%, and diabetes 5% and 10%, respectively (4). In addition, adverse lifestyle patterns are also common, with high levels of excess weight (49% in women and 67% in men), physical inactivity (38% and 24%), and smoking (19% and 27%), while only 21% of both groups show good adherence to the Mediterranean diet (5). These patterns are notable given that Mediterranean populations have traditionally benefited from healthier lifestyles (6), although these advantages appear to be declining, particularly among socioeconomically disadvantaged groups (7,8).

Educational attainment is a key social determinant of health and has been consistently associated with cardiovascular risk and related behaviors (9). Individuals with lower educational levels tend to exhibit less favorable cardiovascular risk profiles and poorer adherence to healthy lifestyles (10,11). These disparities contribute to widening health gaps and highlight the need to examine how cardiovascular risk varies by education and between women and men.

Most research on socioeconomic gradients in cardiovascular risk originates from non-Mediterranean contexts or relies on self-reported data (12,13,14), and few studies have examined comprehensive clinical and lifestyle indicators in population-based samples from Southern Europe (15,16). Addressing this gap is important to better understand how cardiovascular risk is distributed across educational groups in Mediterranean settings.

The objective of this study was to examine the prevalence of cardiovascular risk factors and lifestyle behaviors by educational level in men and women in a population-based sample from a Mediterranean setting.

## Methods

### Study design and population

We conducted a cross-sectional analysis using baseline data from a population-based sample recruited for a randomized controlled trial in Girona and its surrounding areas (north-eastern Spain). The original trial aimed to validate a cardiovascular risk self-screening method and included individuals aged 35–74 years without prior cardiovascular disease. Participants were randomly selected from the reference population and provided written informed consent. The study was approved by the Clinical Research Ethics Committee of the Parc de Salut Mar (CEIC-PSMAR, #2014/5815/I) and registered at ClinicalTrials.gov (#NCT02373319).

The recruitment process has been described in detail elsewhere (4), including the full participant flow. In brief, 955 individuals were initially recruited. Participants with missing data on educational attainment (n = 12), the main exposure variable, were excluded from the present analysis. The final analytical sample therefore comprised 943 individuals with complete baseline data. The sample size for the original trial was estimated at 900 participants to achieve a kappa index of 0.8 (95% confidence interval [CI]) when comparing the self-screening method with a supervised (gold standard) cardiovascular risk assessment. As the present study is a secondary cross-sectional analysis of baseline data, no additional sample size calculation was performed (17).

### Data collection

Trained nurses collected data using standardized procedures. Sociodemographic characteristics (including educational attainment), lifestyle behaviors (smoking status, physical activity, dietary habits), and clinical variables were obtained through self-administered questionnaires and direct measurements. Educational attainment was classified into three categories: (1) primary education (no formal schooling or completion of primary education, typically corresponding to up to 6–8 years of schooling), (2) secondary education (completion of lower and/or upper secondary education, typically up to 12–16 years of schooling), and (3) higher education (completion of a university degree or equivalent). Anthropometric measurements included weight, height, and waist circumference; body mass index (BMI) was calculated as weight (kg)/height^2^ (m^2^). Blood pressure was measured twice after a 5-minute rest using an automated sphygmomanometer, and the lower value was recorded. Hypertension was defined as either self-reported diagnosis, current antihypertensive treatment, or systolic/diastolic blood pressure ≥140/90 mmHg.

Adherence to the Mediterranean diet was assessed using the validated 14-item Mediterranean Diet Adherence Score (MEDAS), ranging from 0 (low adherence) to 14 (high adherence) (5,18). Physical activity was evaluated with the REGICOR questionnaire, which estimates energy expenditure in leisure-time activities based on type, frequency, duration, and intensity. Assigned metabolic equivalent of task (MET) values were walking (4), brisk walking (5), gardening (5), walking trails (6), climbing stairs (8), and sports (10) (5,19).

### Laboratory measurements

Fasting blood samples (10–14 h) were collected in <60 seconds and stored at –80 °C. Total cholesterol and high-density lipoprotein (HDL) cholesterol were measured by enzymatic and direct methods (ABX-Horiba, Montpellier, France). Low-density lipoprotein (LDL) cholesterol was calculated using the Friedewald formula when triglycerides were <300 mg/dL. HbA1c was determined by colorimetry and latex agglutination (ABX-Horiba).

Hypercholesterolemia was defined as self-reported diagnosis, lipid-lowering treatment, total cholesterol ≥190 mg/dL, or LDL cholesterol ≥115 mg/dL. Diabetes was defined as self-reported diagnosis, antidiabetic treatment, HbA1c ≥6.5%, or fasting glycemia ≥126 mg/dL.

### Statistical analysis

Analyses were stratified by sex and educational level. Continuous variables were summarized as means (SD) or medians (IQR), and categorical variables as frequencies. Differences across educational groups were assessed using chi-square tests for categorical variables and ANOVA or Kruskal–Wallis tests for continuous variables, as appropriate. Linear trends were evaluated using p for trend.

Associations between education and cardiovascular risk factors or lifestyle behaviors were examined using age-adjusted regression models: linear regression for continuous outcomes reporting β coefficients and 95% CI, generalized linear models for skewed continuous variables reporting risk ratios and logistic regression for binary outcomes reporting odds ratio (OR) and 95% CI. As a sensitivity analysis, we repeated the analyses in a subsample of women with menopause.

All tests were two-sided, with *p*-value <0.05 considered statistically significant. Analyses were performed using R (version 4.5.2; R Foundation for Statistical Computing, Vienna, Austria) (20).

## Results

The study included 943 participants (52.3% women) with a mean (SD) age of 50 (10) years. Age decreased progressively with higher educational attainment in both sexes.

Among men, lower educational attainment was consistently associated with less favorable cardiovascular profiles. Those with only primary education showed significantly higher LDL cholesterol levels and BMI, and a higher prevalence of hypercholesterolemia, diabetes, and overweight/obesity compared with men with secondary or university education, along with a higher proportion of treated individuals for these conditions. Adherence to the Mediterranean diet increased steadily with educational level (Table 1).

**Table 1:** Cardiovascular risk factors in men by educational attainment.


	PRIMARY EDUCATION (N = 100)	SECONDARY EDUCATION (N = 199)	UNIVERSITY EDUCATION (N = 151)	*p*-VALUE	*p* FOR TREND

Age (years), mean (SD)	53 (11)	49 (10)	49 (10)	0.005	0.013

Smoker, n (%)	34 (34.0)	52 (26.1)	37 (24.7)	0.233	—

Systolic blood pressure (mmHg), mean (SD)	122 (14)	118 (14)	117 (16)	0.062	0.058

Diastolic blood pressure (mmHg), mean (SD)	78 (9)	77 (10)	77 (10)	0.637	—

Hypertension, n (%)	46 (46.9)	60 (30.5)	54 (36.0)	0.021	0.152

Treated hypertension*, n (%)	21 (45.6)	23 (38.3)	22 (40.7)	0.083	—

LDL cholesterol (mg/dL), mean (SD)	150 (37)	137 (35)	131 (31)	0.002	<0.001

HDL cholesterol (mg/dL), mean (SD)	49 (11)	50 (10)	50 (10)	0.846	—

Hypercholesterolemia, n (%)	90 (90.0)	155 (77.9)	115 (77.7)	0.025	0.027

Treated hypercholesterolemia*, n (%)	13 (14.4)	12 (7.7)	12 (10.4)	0.116	—

Glycemia (mg/dL), median (IQR)	97 [90;105]	93 [89;100]	93 [88;100]	0.098	—

Diabetes, n (%)	17 (17.2)	15 (7.6)	13 (8.8)	0.029	0.059

Treated diabetes*, n (%)	11 (64.7)	6 (40.0)	5 (38.5)	0.012	0.012

Body mass index (kg/m^2^), mean (SD)	28.1 (4.7)	26.6 (3.6)	27.1 (4.2)	0.019	0.081

Overweight/Obesity, n (%)	76 (76.0)	121 (60.8)	99 (65.6)	0.033	0.156

Mediterranean diet score, mean (SD)	6.3 (1.8)	6.8 (2.0)	7.3 (2.1)	0.001	<0.001

Adherence to Mediterranean diet score (≥9 points), n (%)	13 (13.0)	41 (20.6)	42 (27.8)	0.018	0.005

Energy expenditure in physical activity (kcal/day), median (IQR)	2273 [923;4443]	2490 [1073;3930]	1944 [1073;3360]	0.304	—

Physical inactivity, n (%)	30 (30.0)	42 (21.1)	37 (24.5)	0.237	—


IQR: Interquartile range. SD: Standard deviation. *For individuals diagnosed.

**Figure 1:**
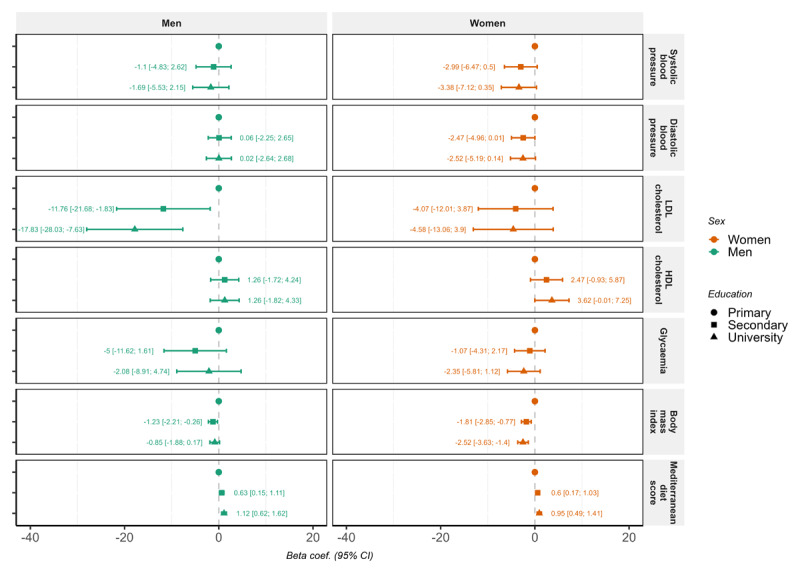
Age-adjusted associations between educational attainment and levels of cardiovascular risk factors in men and women. Estimates represent β coefficients and 95% CIs from linear regression models adjusted for age. Educational attainment is categorized as primary, secondary, and university education (reference group: primary education). Positive β coefficients indicate higher levels of the risk factor compared with the reference group.

**Figure 2:**
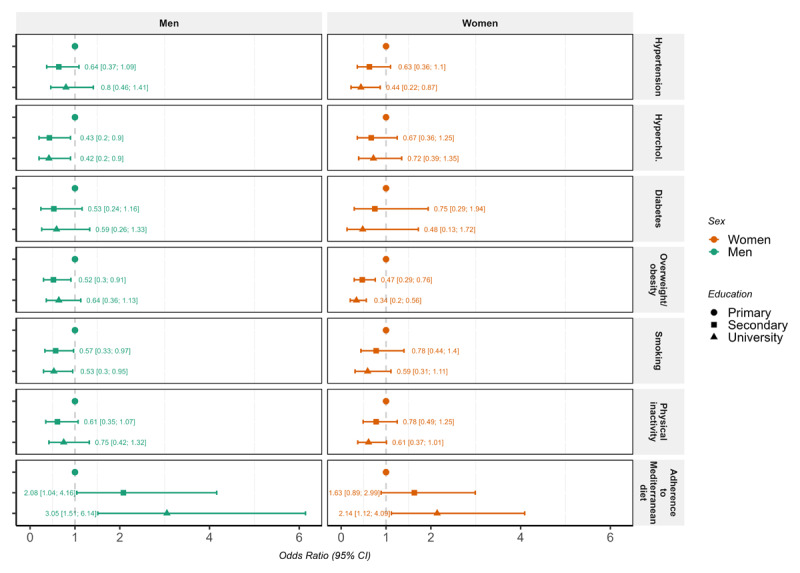
Age-adjusted associations between educational attainment and prevalence of cardiovascular risk factors in men and women. Estimates are odds ratios (ORs) and 95% CIs obtained from logistic regression models adjusted for age. Educational attainment is categorized as primary, secondary, and university education (reference group: primary education). ORs <1 indicate lower odds of the outcome compared with the reference group, whereas ORs >1 indicate higher odds.

In age-adjusted analyses, men with secondary and university education had significantly lower LDL cholesterol concentrations compared with those with primary education [β (95% CI) = –11.76 (–21.68 to –1.83) and β = –17.83 (–28.03 to –7.63), respectively]. They also had lower odds of hypercholesterolemia [OR (95% CI) = 0.43 (0.20–0.90) and OR = 0.42 (0.20–0.90)] and were less likely to smoke [OR = 0.57 (0.33–0.97) and OR = 0.53 (0.30–0.95), respectively]. Mediterranean diet adherence increased with education, both in score [β = 0.63 (0.15–1.11) for secondary education and β = 1.12 (0.62–1.62) for university education] and in high adherence [OR = 2.08 (1.04–4.16) and OR = 3.05 (1.51–6.14), respectively] (Figures 1 and 2).

Among women, lower educational attainment was also associated with more adverse cardiovascular risk factor profiles. Women with only primary education had significantly higher systolic and diastolic blood pressure, LDL cholesterol, glycemia, and BMI, and lower adherence to the Mediterranean diet compared to those with university education. The prevalence of overweight/obesity, hypercholesterolemia, and diabetes also declined progressively with increasing educational level, along with a higher proportion of treated individuals for these conditions (Table 2). In age-adjusted models, BMI was significantly lower among women with secondary and university education compared to those with primary education [β = –1.81 (–2.58 to –0.77) and β = –2.52 (–3.63 to –1.40)], while the OR of overweight/obesity was markedly reduced [OR = 0.47 (0.29–0.76) and OR = 0.34 (0.20–0.56), respectively] (Figures 1 and 2). Women with university education also had significantly lower risk of hypertension [OR = 0.44 (0.22–0.87)]. The prevalence of physical inactivity tended to decrease with education, and, although the association was not statistically significant, women with university education showed lower inactivity prevalence [OR = 0.61 (0.37–1.01)]. HDL cholesterol was marginally higher in women with university education [β = 3.62 (–0.01 to 7.25)]. Mediterranean diet adherence scores were higher in women with secondary and university education [β = 0.60 (0.17–1.03) and β = 0.95 (0.49–1.41), respectively]. However, only women with university education showed higher prevalence of adherence to the Mediterranean diet [OR = 2.14 (1.12–4.09)]. Results from the sensitivity analysis in women with menopause were consistent with those observed in the overall sample of women (Supplementary Tables 1–3).

**Table 2:** Cardiovascular risk factors in women by educational attainment.


	PRIMARY EDUCATION (N = 116)	SECONDARY EDUCATION (N = 197)	UNIVERSITY EDUCATION (N = 180)	*p*-VALUE	*p* FOR TREND

Age (years), mean (SD)	55 (11)	52 (10)	46 (8)	<0.001	<0.001

Smoker, n (%)	25 (20.9)	36 (18.3)	30 (16.7)	0.661	—

Systolic blood pressure (mmHg), mean (SD)	111 (17)	107 (17)	101 (12)	<0.001	<0.001

Diastolic blood pressure (mmHg), mean (SD)	75 (11)	72 (11)	69 (9)	<0.001	<0.001

Hypertension, n (%)	36 (31.0)	38 (19.4)	17 (9.6)	<0.001	<0.001

Treated hypertension*, n (%)	22 (61.1)	16 (42.1)	8 (47.1)	<0.001	0.001

LDL cholesterol (mg/dL), mean (SD)	138 (33)	132 (32)	126 (28)	0.014	0.003

HDL cholesterol (mg/dL), mean (SD)	58 (14)	60 (12)	60 (13)	0.464	—

Hypercholesterolemia, n (%)	97 (83.6)	146 (74.5)	120 (67.0)	0.006	0.002

Treated hypercholesterolemia*, n (%)	18 (18.6)	16 (11.0)	6 (5.0)	0.001	<0.001

Glycemia (mg/dL), median (IQR)	92 [84;98]	90 [84;97]	87 [83;93]	0.008	0.003

Diabetes, n (%)	9 (8.1)	10 (5.2)	4 (2.3)	0.077	0.024

Treated diabetes*, n (%)	7 (77.8)	5 (50.0)	1 (25.0)	0.014	0.005

Body mass index (kg/m^2^), mean (SD)	27.9 (5.4)	25.9 (4.5)	24.6 (4.2)	<0.001	<0.001

Overweight/Obesity, n (%)	81 (69.8)	99 (50.3)	68 (37.8)	<0.001	<0.001

Mediterranean diet score, mean (SD)	6.7 (1.9)	7.2 (2.0)	7.3 (1.8)	0.018	0.011

Adherence to Mediterranean diet score (≥9 points), n (%)	19 (16.4)	44 (22.3)	42 (23.3)	0.325	—

Energy expenditure in physical activity (kcal/day), median (IQR)	1762 [846; 2825]	1809 [917; 2821]	1599 [811; 2481]	0.458	—

Physical inactivity, n (%)	52 (44.8)	77 (39.1)	61 (33.9)	0.165	—


IQR: Interquartile range. SD: Standard deviation.

## Discussion

In this population-based Mediterranean sample, educational attainment was related to healthier behaviors and lower cardiovascular risk factors in both sexes. The sex-stratified analysis revealed that men primarily showed differences in LDL cholesterol, hypercholesterolemia, Mediterranean diet adherence, and smoking prevalence. At the same time, women showed differences across overweight/obesity, blood pressure/hypertension, HDL cholesterol, physical activity, and diet. Taken together, these findings suggest that educational attainment is associated with a broader range of cardiovascular health indicators among women than among men, spanning both biomedical and lifestyle domains. Our results underscore the importance of equity-focused prevention strategies tailored by sex and educational level (21).

### Education and Cardiovascular Risk Pathways

Our findings align with the view of educational attainment as a key social determinant of health that shapes cardiovascular risk through multiple, interlinked pathways (22). At the structural level, education influences employment opportunities, income, and material conditions. These, in turn, affect access to healthy foods, stable housing, and healthcare, factors that provide the broader context in which cardiovascular risk accumulates over the life course (12). At the intermediate level, education is associated with increased health literacy, psychosocial resources, and decision-making capabilities that facilitate engagement with preventive care and adoption of healthy behaviors, including diet quality and smoking avoidance (23,24). Moreover, evidence shows that the cardiovascular benefits of Mediterranean-style eating and healthy lifestyles tend to be patterned by education or related socioeconomic markers (25). At the individual level, these upstream advantages translate into measurable differences in biomedical risk factors such as adiposity, blood pressure, and lipid profile (12). Despite their relevance as mediators of socioeconomic differences, individual-level factors account for only a small proportion of the overall socioeconomic inequity in health (9). Thus, healthy-lifestyle promotion alone is unlikely to substantially reduce socioeconomic disparities in cardiovascular health, underscoring the need for complementary strategies that address the upstream social determinants of health (26).

### Sex Differences and Underlying Mechanisms

Building on these pathways, our results suggest that higher educational attainment is associated with more favorable cardiovascular risk profiles among women than among men, indicating that education-related pathways may be particularly relevant in women. One plausible mechanism is health literacy, for which educational level is a key determinant, conferring greater capacity for risk recognition and adoption of preventive behaviors (27). Limited health literacy has been linked to increased cardiovascular risk and remains more prevalent among women from historically marginalized groups and with lower educational attainment (28,29). This may contribute to the wider differences observed in biomedical risk factors among women across educational levels.

In addition, gender roles and caregiving responsibilities may further amplify the impact of education on cardiometabolic risk in women. Caregiving burden—more common among women—has been associated with poorer physical activity, diet quality, and stress regulation, whereas resources and greater agency linked to higher education may mitigate these effects (30,31). This provides an additional pathway through which education may buffer caregiving-related risks, contributing to steeper gradients in adiposity and blood pressure among women (32).

### Public Health and Policy Implications

Our findings have clear implications for public health practice. The stronger educational gradients observed among women support the need for prevention strategies that integrate both equity-focused and sex-specific approaches. Strengthening primary care prevention and cardiometabolic screening is essential to reduce undiagnosed and undertreated risk factors, particularly in socially disadvantaged groups (33,34).

Beyond clinical interventions, reducing cardiovascular inequalities requires sustained action on structural determinants, including educational opportunities, employment conditions, and neighborhood environments that shape daily living conditions (12). Given the patterns observed, women with lower educational attainment may face intersecting constraints—such as socioeconomic disadvantage and caregiving responsibilities—that increase cardiometabolic vulnerability (13,35,36).

In sum, a dual strategy is warranted: (1) reinforcing equity-informed clinical and community prevention and (2) addressing the structural drivers of educational inequalities. These findings highlight the importance of gender-responsive approaches across public health practice and policy.

### Strengths and Limitations

The consistency of our age-adjusted associations suggests that the observed educational gradients do not merely reflect higher educational attainment among younger—and healthier—participants. Nonetheless, residual confounding cannot be ruled out. Income, occupation, neighborhood context, and life-course exposures (e.g., early-life adversity) were not included and may mediate or confound the observed associations. Selective survival and participation may also bias estimates in cross-sectional designs. Although our population-based sampling frame and standardized recruitment procedures help mitigate this concern, it cannot be completely excluded. Moreover, the cross-sectional nature of the study precludes establishing temporality. Educational attainment is typically completed before age 35, which was the minimum recruitment age in our sample, whereas the development of many cardiovascular risk factors tends to occur later in adulthood, which reduces the likelihood of reverse causation. In addition, given the number of outcomes examined across sex and educational groups, some associations may be due to chance and should be interpreted cautiously. Treatment was also included in the definition of cardiovascular risk factors. As a result, observed differences across educational levels should be understood in the context of underlying disease prevalence rather than as independent differences in access to or use of care.

A major strength of this study is the analysis of a relatively large, population-based sample from Southern Europe, using standardized and objective measurements of key cardiovascular risk factors, together with validated instruments to assess diet and physical activity. In addition, stratification of the analyses by sex represents a further strength, as it allowed us to identify sex-specific patterns that would likely have been obscured in pooled analyses.

## Conclusion

In this Mediterranean population, higher educational attainment is consistently associated with healthier lifestyles and more favorable cardiovascular risk profiles, with marked sex-specific patterns. These findings extend previous evidence by showing that socioeconomic inequalities in cardiovascular risk persist, and may even be pronounced, in settings traditionally considered protective, such as Mediterranean populations. The broader gradients observed among women suggest that educational attainment may operate through multiple, interacting pathways shaping both behavioral and biological risks, reinforcing the need to consider sex-specific mechanisms in the study of health inequalities. Policies and interventions that improve access to healthy lifestyles, strengthen sex-specific prevention, and address upstream social conditions are essential to effectively reduce these disparities.

## Data Accessibility Statement

The original contributions presented in this study are included in the article; further inquiries can be directed to the corresponding author.

## Additional File

The additional file for this article can be found as follows:

10.5334/gh.1574.s1 Supplementary Files. Tables 1 to 3.

## References

[1] World Health Organization. WHO mortality database. Geneva: World Health Organization; 2024. https://www.who.int/data/data-collection-tools/who-mortality-database (January 2026, date last accessed).

[2] Flores-Mateo G, Grau M, O’Flaherty M, Ramos R, Elosua R, Violan-Fors C, Quesada M, Marti R, Sala J, Marrugat J, Capewell S. Analyzing the coronary heart disease mortality decline in a Mediterranean population: Spain 1988–2005. Revista Española de Cardiología. 2011;64:988–996. DOI: 10.1016/j.recesp.2011.05.033 21962958

[3] World Health Organization. World health statistics 2017: monitoring health for the SDGs, sustainable development goals. Geneva: World Health Organization; 2017. https://www.who.int/publications/i/item/world-health-statistics-2017 (January 2026, date last accessed).

[4] Barroso M, Zomeño MD, Díaz JL, Pérez-Fernández S, Martí-Lluch R, Cordón F, Ramos R, Cabezas C, Salvador G, Castell C, Schröder H. Control of cardiovascular risk factors with tailored recommendations: A randomized controlled trial. Preventive Medicine. 2020;141:106302. DOI: 10.1016/j.ypmed.2020.106302 33144141

[5] Barroso M, Zomeño MD, Díaz JL, Pérez S, Martí-Lluch R, Cordón F, Ramos R, Cabezas C, Salvador G, Castell C, Schröder H. Efficacy of tailored recommendations to promote healthy lifestyles: a post hoc analysis of a randomized controlled trial. Translational Behavioral Medicine. 2021;11:1548–1557. DOI: 10.1093/tbm/ibab035 33837787

[6] Estruch R, Ros E, Salas-Salvadó J, Covas MI, Corella D, Arós F, Gómez-Gracia E, Ruiz-Gutiérrez V, Fiol M, Lapetra J, Lamuela-Raventos RM. Primary prevention of cardiovascular disease with a Mediterranean diet supplemented with extra-virgin olive oil or nuts. New England Journal of Medicine. 2018;378:e34. DOI: 10.1056/NEJMoa1800389 29897866

[7] Bach-Faig A, Fuentes-Bol C, Ramos D, Carrasco JL, Roman B, Bertomeu IF, Cristia E, Geleva D, Serra-Majem L. The Mediterranean diet in Spain: adherence trends during the past two decades using the Mediterranean Adequacy Index. Public Health Nutrition. 2011;14:622–628. DOI: 10.1017/S1368980010002752 21029509

[8] Obeid C, Oenema A, Jaalouk D, Kremers SPJ, Gubbels JS. Determinants of adherence to the Mediterranean diet among adults in Mediterranean countries: a systematic literature review. Public Health Nutrition. 2025;28:e194. DOI: 10.1017/S1368980025101432 41199361 PMC12722083

[9] Dégano IR, Marrugat J, Grau M, Salvador-González B, Ramos R, Zamora A, Martí R, Elosua R. The association between education and cardiovascular disease incidence is mediated by hypertension, diabetes, and body mass index. Scientific Reports. 2017;7:12370. DOI: 10.1038/s41598-017-10775-3 28959022 PMC5620039

[10] Stringhini S, Carmeli C, Jokela M, Avendaño M, Muennig P, Guida F, Ricceri F, d’Errico A, Barros H, Bochud M, Chadeau-Hyam M. Socioeconomic status and the 25 × 25 risk factors as determinants of premature mortality: A multicohort study and meta-analysis of 1.7 million men and women. Lancet. 2017;389:1229–1237. DOI: 10.1016/S0140-6736(16)32380-7 28159391 PMC5368415

[11] Mackenbach JP, Kulhánová I, Artnik B, Bopp M, Borrell C, Clemens T, Costa G, Dibben C, Kalediene R, Lundberg O, Martikainen P. Changes in mortality inequalities over two decades: register based study of European countries. British Medical Journal. 2016;353:i1732. DOI: 10.1136/bmj.i1732 27067249 PMC4827355

[12] Bann D, Wright L, Hughes A, Chaturvedi N. Socioeconomic inequalities in cardiovascular disease: A causal perspective. Nature Reviews Cardiology. 2024;21:238–249. DOI: 10.1038/s41569-023-00941-8 37821646

[13] Xia M, An J, Safford MM, Colantonio LD, Sims M, Reynolds K, et al. Cardiovascular risk associated with social determinants of health at individual and area levels. JAMA Netw Open. 2024;7:e248584. DOI: 10.1001/jamanetworkopen.2024.8584 38669015 PMC11053380

[14] Kislaya I, Perelman J, Tolonen H, Nunes B. Do self-reported data accurately measure health inequalities in risk factors for cardiovascular disease? International Journal of Public Health. 2019;64:721–729. DOI: 10.1007/s00038-019-01232-1 30906957

[15] Grau M, Elosua R, Cabrera de León A, Guembe MJ, Baena-Díez JM, Vega Alonso T, Felix FJ, Zorrilla B, Rigo F, Lapetra J, Gavrila D. Cardiovascular risk factors in Spain in the first decade of the 21st Century, a pooled analysis with individual data from 11 population-based studies: the DARIOS study. Revista Española de Cardiología. 2011; 64:295–304. DOI: 10.1016/j.recesp.2010.11.005 21397375

[16] Gullón P, Díez J, Cainzos-Achirica M, Franco M, Bilal U. Social inequities in cardiovascular risk factors in women and men by autonomous regions in Spain. Gaceta Sanitaria. 2021;35:326–332. DOI: 10.1016/j.gaceta.2020.04.014 32674863 PMC7985704

[17] Barroso M, Pérez-Fernández S, Vila MM, Zomeño MD, Martí-Lluch R, Cordon F, Ramos R, Elosua R, Degano IR, Fito M, Cabezas C. Validity of a method for the self-screening of cardiovascular risk. Clinical Epidemiology. 2018;10:549–560. DOI: 10.2147/CLEP.S158358 29785141 PMC5953309

[18] Schröder H, Fitó M, Estruch R, Martínez-González MA, Corella D, Salas-Salvadó J, Lamuela-Raventós R, Ros E, Salaverría I, Fiol M, Lapetra J. A short screener is valid for assessing Mediterranean diet adherence among older Spanish men and women. Journal of Nutrition. 2011;141:1140–1145. DOI: 10.3945/jn.110.135566 21508208

[19] Molina L, Sarmiento M, Peñafiel J, Donaire D, Garcia-Aymerich J, Gomez M, Ble M, Ruiz S, Frances A, Schröder H, Marrugat J. Validation of the Regicor short physical activity questionnaire for the adult population. PLoS One. 2017;12:e0168148. DOI: 10.1371/journal.pone.0168148 28085886 PMC5234797

[20] Team RC. RA Language and environment for statistical computing. Vienna: Austria: R Foundation for Statistical Computing; 2025. https://www.R-project.org/

[21] Backholer K, Peters SAE, Bots SH, Peeters A, Huxley RR, Woodward M. Sex differences in the relationship between socioeconomic status and cardiovascular disease: a systematic review and meta-analysis. Journal of Epidemiology and Community Health. 2017;71:550–557. DOI: 10.1136/jech-2016-207890 27974445

[22] Institute for Health Metrics and Evaluation (IHME). Social determinants of health and cardiovascular disease: state of the evidence. Seattle, WA: IHME; 2023.

[23] Lou SP, Han D, Kuczmarski MF, Evans MK, Zonderman AB, Crews DC. Health literacy, numeracy, and dietary approaches to stop hypertension accordance among hypertensive adults. Health Education & Behavior. 2023;50:49–57. DOI: 10.1177/10901981221079742 35272527 PMC11681931

[24] Zomeño MD, Álamo-Junquera D, Pericas C, Vilaplana-Carnerero C, Domínguez À, Toledo D, Soldevila N, Pagès-Fernández C, Redondo A, Tor-Roca A, Grau M. Cardiovascular disease prevention by personalized health promotion considering educational attainment. Scientific Reports. 2026;16:6604. DOI: 10.1038/s41598-026-36654-4 41611770 PMC12913767

[25] Reig-García G, Martínez-Sancho J, Baltasar-Bagué A, Mateu-Figueras G, Buxó M, Martí-Lluch R, Zacarías-Pons L, Puig J, Ramos-Blanes R, Fernández-Real JM, Garre-Olmo J. Adherence to Mediterranean diet and physical activity practice in general population: an intersectional analysis of inequalities by sex and economic status. Frontiers in Public Health. 2025;13:1727223. DOI: 10.3389/fpubh.2025.1727223 41473726 PMC12745204

[26] Zhang YB, Chen C, Pan XF, Guo J, Li Y, Franco OH, Liu G, Pan A. Associations of healthy lifestyle and socioeconomic status with mortality and incident cardiovascular disease: two prospective cohort studies. British Medical Journal. 2021;373:n604. DOI: 10.1136/bmj.n604 33853828 PMC8044922

[27] Stormacq C, Van den Broucke S, Wosinski J. Does health literacy mediate the relationship between socioeconomic status and health disparities? Integrative review. Health Promotion International. 2019;34:e1–e17. DOI: 10.1093/heapro/day062 30107564

[28] Beasant B, Anderson K, Lee G, Lotfaliany M, Tembo M, McCoombe S, Vaughan V, Pasco JA, Hosking SM. Health literacy and primary prevention of cardiovascular disease: A scoping review. Public Health Reports. 2025;140:342–357. DOI: 10.1177/00333549251322649 40488427 PMC12149170

[29] Metlock FE, Kwapong YA, Evans C, Ouyang P, Vaidya D, Kwapong YA, Hladek MD, Ouyang P, Hall JL, Sharma G, Commodore-Mensah Y. Cardiovascular health literacy among women of reproductive age in the SAFE HEART Study: An American Heart Association Research Goes Red Initiative. Health Literacy and Communication Open. 2025;3:2553042. DOI: 10.1080/28355245.2025.2553042

[30] Lindt N, van Berkel J, Mulder BC. Determinants of overburdening among informal carers: a systematic review. BMC Geriatrics. 2020;20:304. DOI: 10.1186/s12877-020-01708-3 32847493 PMC7448315

[31] Ahn S, Son EH, Mogos MF, Muchira JM, Sheng Y, Park C, Lee LJ. Patterns of lifestyle risk behaviors for cardiovascular disease in family caregivers: a latent class analysis. Frontiers in Public Health. 2025;13:1593898. DOI: 10.3389/fpubh.2025.1593898 40600154 PMC12209217

[32] Skinner MS, Sogstad M. Social and gender differences in informal caregiving for sick, disabled, or elderly persons: A cross-sectional study. SAGE Open Nursing. 2022;8:23779608221130585. DOI: 10.1177/23779608221130585 36238939 PMC9551342

[33] Lawrence WR, Hong HG, Williams F, Dyer Z, Bergeron NQ, Brewer LC, Chen Y, Crittendon DR, Freedman ND, Haas CB, Jackson SS. Manifestations of structural racism and inequities in cardiovascular health across US neighborhoods. Journal of the American Medical Association. 2025;6:e253864. DOI: 10.1001/jamahealthforum.2025.3864 PMC12579350 41171261

[34] OECD. Gender equality in a changing world: taking stock and moving forward. Paris: OECD Publishing; 2025. DOI: 10.1787/e808086f-en

[35] Vogel B, Acevedo M, Appelman Y, Bairey Merz CN, Chieffo A, Figtree GA, Guerrero M, Kunadian V, Lam CS, Maas AH, Mihailidou AS. The Lancet women and cardiovascular disease commission: Reducing the global burden by 2030. Lancet. 2021;397:2385–2438. DOI: 10.1016/S0140-6736(21)00684-X 34010613

[36] Bugiardini R, Rahaman T, Manfrini O, Maas A, Bergami M, Badimon L, Mendieta G, Vavlukis M, Merkely B, Vasiljevic Z, Gale CP. Diet and sex inequities in ischemic heart disease mortality across Europe: Findings from the global burden of disease study. Cardiovascular Research. 2025;121:2432–2446. DOI: 10.1093/cvr/cvaf176 41181878 PMC12687866

