# Supplementary material for: Lifestyle and Cardiovascular Risk Factors by Educational Attainment in a Mediterranean Setting: A Cross-Sectional Analysis of a Population-Based Sample
Source: Glob Heart. 2026 Jul 14;21(1):55. doi: 10.5334/gh.1574 (PMC13378425; doi:10.5334/gh.1574)
Supplement: Supplementary Files. — Tables 1 to 3. [file gh-21-1-1574-s1.pdf]

Lifestyle and Cardiovascular Risk Factors by Educational Attainment in a Mediterranean Setting: A Cross-Sectional Analysis of a Population-Based Sample

Supplementary material

Dolores Álamo-Junquera[1,2], Carles Vilaplana-Carnerero[2,3,4], Constança Pagès-Fernández[2], Diana Toledo[2,4], Núria Soldevila[2,4], Alba Tor-Roca,[2,5,6] Àngela Domínguez[2,4], María Grau[2,4,7*]

[1] Catalan Institute of Health, Sabadell, Spain

[2] Department of Medicine, School of Medicine and Health Sciences, University of Barcelona, Barcelona, Spain

[3] Service for the Promotion of Quality and Bioethics, General Directorate of Health Planning and Regulation, Department of Health, Government of Catalonia, Barcelona, Spain

[4] Biomedical Research Consortium in Epidemiology and Public Health (CIBERESP), Madrid, Spain

[5] Jordi Gol i Gurina Primary Health Care Research Institute Foundation (IDIAPJGol), Barcelona, Spain

[6] Research Institute for Nutrition and Food Safety (INSA-UB), Santa Coloma de Gramenet, Spain

[7] Instituto de Salud Global de Barcelona (ISGlobal), Barcelona, Spain

Supplementary Table 1. Sensitivity analysis for women with menopause

	**Primary Education (N=65)**	**Secondary Education (N=109)**	**University Education (N=44)**	**p-value**	**p for trend**
Age (years), mean (SD)	62 (7)	59 (8)	57 (6)	<0.001	<0.001
Smoker, n (%)	10 (14.1)	18 (16.5)	7 (15.9)	0.911	--
Systolic blood pressure (mmHg), mean (SD)	119 (17)	112 (18)	108 (11)	0.002	0.002
Diastolic blood pressure (mmHg), mean (SD)	79 (11)	74 (10)	73 (8)	0.005	0.003
Hypertension, n (%)	40 (38.5)	32 (28.7)	12 (23.8)	0.225	--
Treated hypertension*, n (%)	17 (42.5)	14 (43.8)	6 (50.0)	0.116	--
LDL cholesterol (mg/dL), mean (SD)	150 (31)	139 (34)	136 (27)	0.066	--
HDL cholesterol (mg/dL), mean (SD)	60 (15)	62 (13)	63 (13)	0.556	--
Hypercholesterolemia, n (%)	61 (93.8)	93 (85.2)	38 (86.4)	0.220	--
Treated hypercholesterolemia*, n (%)	15 (24.6)	15 (16.1)	4 (10.5)	0.108	
Glycemia (mg/dL), median [IQR]	94 [87;102]	92 [86;99]	91 [85;95]	0.174	--
Diabetes, n (%)	11 (12.9)	8 (4.7)	2 (4.5)	0.128	--
Treated diabetes*, n (%)	6 (54.5)	2 (25.0)	1 (50.0)	0.063	--
Body mass index (kg/m^2), mean (SD)	28.6 (5.1)	26.2 (4.3)	26.2 (4.9)	0.005	0.003
Overweight / Obesity, n (%)	15 (23.1)	49 (45.0)	20 (45.5)	0.009	0.009
Mediterranean diet score, mean (SD)	6.8 (1.9)	7.6 (2.0)	8.0 (1.9)	0.004	0.002
Adherence to Mediterranean diet score (≥9 points), n (%)	11 (16.9)	31 (28.4)	15 (34.1)	0.100	--
Energy expenditure in physical activity (kcal/day), median [IQR]	1865 [1028;2977]	2126 [1161;3387]	1713 [874;2739]	0.411	--
Physical inactivity, n (%)	28 (43.1)	39 (35.8)	17 (38.6)	0.633	--

IQR: Interquartile range. SD: Standard deviation

Supplementary Table 2. Age-adjusted associations between educational attainment and levels of cardiovascular risk factors in women with menopause

	Secondary Education			University Education		
	**β-coefficient**	**95% confidence interval**	**p-value**	**β-coefficient**	**95% confidence interval**	**p-value**
Systolic blood pressure (mmHg)	-4.97	-10.02; 0.08	0.055	-6.06	-12.40; 0.27	0.062
Diastolic blood pressure (mmHg)	-4.52	-7.78; -1.26	0.007	-4.21	-8.30; -0.12	0.045
LDL colesterol (mg/dl)	-10.32	-21.52; 0.88	0.073	-12.13	-26.03; 1.78	0.089
HDL colesterol (mg/dl)	2.57	-2.30; 7.45	0.302	3.35	-2.71; 9.42	0.280
Glycaemia (mg/dl)	-3.00	-7.98; 1.98	0.240	-4.70	-10.90; 1.51	0.140
Body mass index (kg/m^2)	-2.15	-3.59; -0.70	0.004	-2.04	-3.87; -0.22	0.029
Mediterranean diet score (points)	0.94	0.33; 1.54	0.003	1.32	0.55; 2.09	0.001

Supplementary Table 3. Age-adjusted associations between educational attainment and and prevalence of cardiovascular risk factors in women with menopause

	**Secondary education**			**University education**		
	**Odds Ratio**	**95% confidence interval**	**p-value**	**Odds Ratio**	**95% confidence interval**	**p-value**
Hypertension	0.77	0.39; 1.51	0.442	0.68	0.27; 1.67	0.396
Hypercholesterolemia	0.46	0.14; 1.46	0.186	0.55	0.14; 2.14	0.388
Diabetes	0.43	0.13; 1.44	0.172	0.53	0.10; 2.85	0.462
Obesity	0.38	0.19; 0.77	0.007	0.38	0.16; 0.90	0.027
Smoker	0.90	0.36; 2.23	0.816	0.75	0.25; 2.31	0.620
Physical inactivity	0.77	0.40; 1.45	0.416	0.89	0.40; 1.99	0.772
Adherence to Mediterranean diet	2.03	0.93; 4.45	0.077	2.70	1.06; 6.86	0.036
